# Topological properties of a bipartite lattice of domain wall states

**DOI:** 10.1038/s41598-018-35651-6

**Published:** 2018-11-26

**Authors:** F. Munoz, Fernanda Pinilla, J. Mella, Mario I. Molina

**Affiliations:** 1 Departamento de Física, Facultad de Ciencias, Universidad de Chile, Santiago, Chile; 2 Center for the Development of Nanoscience and Nanotechnology (CEDENNA), Santiago, Chile; 3 MSI-Nucleus on Advanced Optics, Facultad de Ciencias, Universidad de Chile, Santiago, Chile

## Abstract

We propose a generalization of the Su-Schrieffer-Heeger (SSH) model of the bipartite lattice, consisting of a periodic array of domain walls. The low-energy description is governed by the superposition of localized states at each domain wall, forming an effective mono-atomic chain at a larger scale. When the domain walls are dimerized, topologically protected edge states can appear, just like in the original SSH model. These new edge states are formed exclusively by soliton-like states and therefore, the new topological states are qualitatively different from the regular SSH edge states. They posses a much longer localization length and are more resistant to on-site disorder, in marked contrast to the standard SSH case.

## Introduction

The last years have witnessed a growing interest on one-dimensional models of non-trivial topological systems. This has been largely favored by the rapid advance of photonics and nano-photonics as an ideal playground to experimentally corroborate theoretical predictions^1–3^. Different experimental setups, such as plasmonic nanoparticles and phononic lattices, have been used as practical realizations of one-dimensional models^4–8^. This emergence of novel experimental escenarios, in turn, has motivated further theoretical research, often on basic aspects or phenomena beyond the scope of common electronic systems, such as non-linearities^9,10^ and Floquet insulators^8,11,12^. The interest on one-dimensional topologically protected modes is not only related to basic understanding, but to the practical implementation in the design of low-loss devices^13,14^.

Perhaps, the most studied one-dimensional model with a non-trivial topology is the Su-Schrieffer-Heeger model of polyacetylene^15^ (SSH). It consists on a tight-binding model for the bipartite lattice, and displays soliton-like localized edge states at domain walls (*i.e*. stacking faults of the bipartite lattice). Recently, this model has been the subject of generalizations in order to observe new phenomena^16,17^. Other one-dimensional models, not restricted to the bipartite lattice, have been proposed and realized, showing novel edge states^6,18–20^.

One of the main purposes behind the focus in simpler models as the SSH is to get insight on more complex systems or materials. For instance, depending on the termination, a non-trivial Zak phase in nanoribbons can arise, implying topologically-protected edge states^21^. Similarly, it was shown that some edges states in 2D systems with a negligible spin-orbit coupling -for instance some terminations of black phosporus- are indeed topologically protected^22^. Also, the natural extension of the SSH model to two dimensions can host different topological phases, even with a zero Berry phase^23^.

In this article we start by reproducing the results of the famous SSH model, Sec. 1, to set the notation and make its generalization easier. In Sec. 2, we introduce a new model, consisting on *N* interacting copies of the SSH model, originated from an array of domain walls. If the lattice of domain walls is dimerized -a bipartite lattice of domain walls-, we recover a SSH-like behavior formed by the superposition of localized modes. Naturally, this model has a non-trivial phase featuring edge states. In Sec. 3 the effect of disorder in the model is studied. Unlike the SSH model, the new edge modes are somewhat robust to on-site disorder.

## The Bipartite Lattice

**Figure 1:**
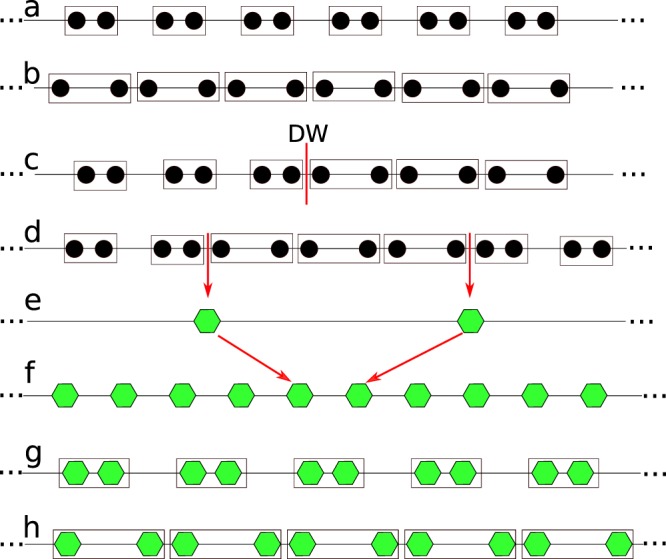
(**a**,**b**) Scheme of the bipartite lattice, Eq. (1), where each cell -enclosed by a frame- has two sites. There are two cases: Intra-cell coupling stronger that the inter-cell coupling or the converse. No specific boundaries are implied for any of the schemes. (**c**) The SSH model: a domain wall defect (DW), see Eq. (4). At the DW a localized state appears, lying in the middle of the bandgap, see Eqs (5 and 6). (**d**,**e**) Two DWs, the length of each segment is exaggerated for ease in visualization. The green hexagon denotes the DW state (low-energy state), see Eq. (14). (**f**) A periodic lattice of DWs. Here, the effective low-energy description is a mono-atomic chain, see Eqs (10–12). (**g**,**h**) The Bipartite Domain Wall model (BDW): Dimerization of the DWs. Now, the low-energy behavior is a bipartite lattice, see Eqs (15–17), just like panels (**a**,**b**). The introduction and study of the BDW model is the subject of this article.

We start with a brief summary of the SSH model of polyacetylene^15^, or more precisely, the tight-binding formulation of the bipartite lattice, see Fig. 1. We assume that the reader has some acquaintance with chiral symmetry, the SSH model and topological states of matter^21,24^. In the optical context, the edge states from this model have been experimentally observed and theoretically explained in photonic superlattices^1,25^.

In a finite bipartite lattice, the two different orderings (see, Fig. 1a,b) acquire a physical meaning. Localized edge states appears when the inter-cell coupling dominates. In this case, the edge states are sub-lattice polarized. It is tempting to call this phase ‘topological’, however in a finite chain the interaction between edge states is small but not negligible, resulting in a finite interaction energy. Nevertheless, the edge states remain sub-lattice polarized and they determine the low-energy phenomena.

A stacking fault on the chain couplings results in a domain wall (DW), Fig. 1c, where a zero-energy localized state appears (called a soliton in the context of polyacetylene). Often, domain walls come into pairs, Fig. 1d. If their distance is not too large, they interact weakly developing low-energy modes. Therefore, the low-energy physics is dominated by DW states, and we can ignore the bulk’s valence and conduction bands, Fig. 1e. A periodic set of DWs and its corresponding periodic lattice of DWs states leads to an effective mono-atomic lattice, Fig. 1f. However, if the array of DWs is dimerized, the low-energy states will be a bipartite lattice, see Fig. 1g,h. Such a lattice resembles the original SSH lattice and may have low-energy, topologically protected edge states. In the remainder of this article we will delve on this possibility.

### Free boundaries

**Figure 2:**
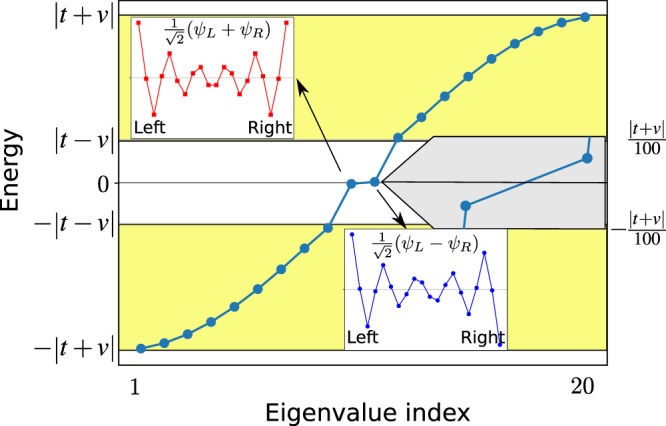
Energy levels and edge states for a finite bipartite lattice in the topological phase (*t* < *v*). *N* = 10 cells (20 sites), $t=1$, $v=\frac{3}{2}$. The insets are the edge states in real space and a zoom to their energy (its scale is on the right border).

The one-dimensional bipartite lattice, see Fig. 1a,b, has the Hamiltonian

1 $${H}_{SSH}=t\,\sum _{j=0}^{N-1}\,{a}_{j}^{\dagger }{b}_{j}+v\,\sum _{j=0}^{N-2}\,{a}_{j+1}^{\dagger }{b}_{j}+H\mathrm{.}c\mathrm{.},$$

where *t* and *v* are the hopping amplitudes and each cell has two equal –but inequivalent– sites *a*, *b* (*e.g*. the sub-lattices). In spite of its simplicity, this system has a very rich physics. In the case of *t* < *v*, it has (almost) zero-energy and sub-lattice polarized edge states solutions. In the limit *N* → ∞ the zero-energy modes have a simple expression

2 $${\psi }_{L}^{\dagger }=\alpha \,\sum _{j=0}^{N-1}\,{(-\mathrm{1)}}^{j}{e}^{-\frac{j}{\varepsilon }}{a}_{j}^{\dagger }$$

3 $${\psi }_{R}^{\dagger }=\beta \,\sum _{j=0}^{N-1}\,{(-\mathrm{1)}}^{N-j}{e}^{-\frac{N-j}{\varepsilon }}{b}_{j}^{\dagger },$$

where *ψ*_*L*_ is localized at the left edge of the chain, with width $\varepsilon =\,\mathrm{log}(\frac{v}{t})$. The state *ψ*_*R*_ is localized at the right edge. *α*, *β* are normalization constants. For a finite chain size, *N*, the interaction between both edge states is small but finite $\langle {\psi }_{L}|{H}_{open}|{\psi }_{R}\rangle \propto {e}^{-\frac{N}{\varepsilon }}$, forming a bonding and anti-bonding pair: $\frac{1}{\sqrt{2}}({\psi }_{L}\pm {\psi }_{R})$, see Fig. 2a,b. In Fig. 2 the finite size effects open a band gap nearly 100 times smaller than the band gap of the bulk system.

The bulk-like states, have energies $E=\pm \,\sqrt{{t}^{2}+{v}^{2}+2tv\,\cos (k)}$, see Fig. 2, the band gap (*i.e*. excluding edge states) is $\mathrm{2|}t-v|\gg 2\varepsilon$, validating our previous statement that bulk states are irrelevant for a low-energy description.

### Domain walls and periodic boundaries

Both bonding schemes of the bipartite lattice, see Fig. 1a,b can coexist next to each other, meeting on a lattice defect called Domain Wall (DW), see Fig. 1c. If each subsystem consist of *N* sites, after imposing periodic boundary conditions, *i.e*. by setting ${a}_{2N}^{\dagger }\equiv {a}_{0}^{\dagger }$, the whole lattice has two DWs, each with a localized state centered on it. The Hamiltonian of the full system is:

4 $${H^{\prime} }_{SSH}=\sum _{j=0}^{N-1}\,(t{a}_{j}^{\dagger }{b}_{j}+v{a}_{j+1}^{\dagger }{b}_{j})+\sum _{j=N}^{2N-1}\,(v{a}_{j}^{\dagger }{b}_{j}+t{a}_{j+1}^{\dagger }{b}_{j})+H\mathrm{.}c\mathrm{.}$$

We can use the previous solutions *ψ*_*L*_, *ψ*_*R*_, Eqs (2 and 3) as an *ansatz* for the DW states at $j=0,N-\frac{1}{2}$:

5 $${\psi }_{a}^{\dagger }=\alpha \sum _{j=0}^{N-1}\,{(-\mathrm{1)}}^{j}{e}^{-\frac{j}{\varepsilon }}{a}_{j}^{\dagger }+\alpha \sum _{j=N}^{2N-1}\,{(-\mathrm{1)}}^{j}{e}^{-\frac{2N-j}{\varepsilon }}{a}_{j}^{\dagger }$$

6 $${\psi }_{b}^{\dagger }=\beta \sum _{j=1}^{N-1}\,{(-\mathrm{1)}}^{j}{e}^{-\frac{N-1-j}{\varepsilon }}{b}_{j}^{\dagger }+\beta \sum _{j=N}^{2N-1}\,{(-\mathrm{1)}}^{j}{e}^{-\frac{j-N}{\varepsilon }}{b}_{j}^{\dagger },$$

where *α*, *β* are normalization constants. These solutions are valid for any value of *t* ≠ *v*. If *v* > *t* (or *ε* > 0), *ψ*_*a*_ is localized at *j* = 0 and *ψ*_*b*_ localized on $j=N+\frac{1}{2}$. A negative value of *ε* just reverses the positions of the DW centers.

## A Lattice of Periodic Domain Walls

### Monospaced and Periodic Domain Walls

A bipartite lattice with *M* mono-spaced DWs –see Fig. 1f– has the following Hamiltonian

7 $${H}_{PDW}=\sum _{m=0}^{M-1}\,({h}_{N}(m)+{h^{\prime} }_{N}(m))$$

8 $${h}_{N}(m)=\sum _{j=2mN}^{\mathrm{(2}m+\mathrm{1)}N-1}\,(t{a}_{j}^{\dagger }{b}_{j}+v{a}_{j+1}^{\dagger }{b}_{j})+H\mathrm{.}c.$$

9 $${h^{\prime} }_{N}(m)=\sum _{j=\mathrm{(2}m+\mathrm{1)}N}^{\mathrm{(2}m+\mathrm{2)}N}\,(v{a}_{j}^{\dagger }{b}_{j}+t{a}_{j+1}^{\dagger }{b}_{j})+H\mathrm{.}c\mathrm{.},$$

where *h*_*N*_, *h*′_*N*_ are SSH-like Hamiltonians, but with different topological phase. The periodicity in *H*_*PDW*_ is two DWs, or 2*N* cells, but the spacing between domain walls is just *N*. This Hamiltonian is quite cumbersome, but if we focus on its low-energy excitations, it can be greatly simplified, by just keeping the superposition of modes *ψ*_*a*_, *ψ*_*b*_, at each domain wall, see Eqs (5 and 6). Using them as a basis, the effective Hamiltonian is:

10 $${H}_{PDW}^{eff}=\sum _{m=0}^{M-1}t^{\prime} \,({\psi }_{a,m}^{\dagger }{\psi }_{b,m}+{\psi }_{a,m+1}^{\dagger }{\psi }_{b,m})+h.\,c.,$$

**Figure 3:**
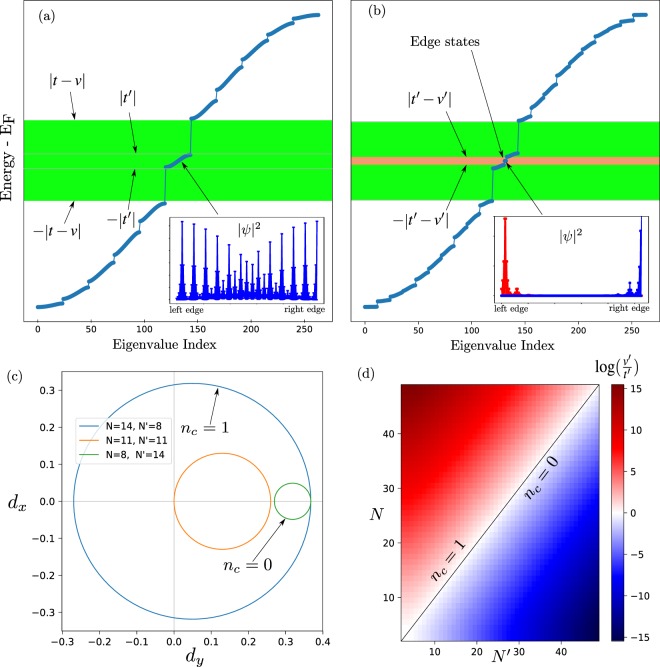
(**a**) Energy levels of an array of *M* = 12 DWs, with a spacing of *N* = 11 cells. The low-energy eigenstates -inside the green region-, are formed by interactions among DW states, see the inset. (**b**) Energy levels of a lattice of DWs, but with an alternate width, *N* = 14, *N*′ = 8, between successive DWs (*M* = 12 replications were used). A new bandgap opens with topologically protected edge states in the middle, see the reddish region and the inset. (**c**) Representation of the effective Hamiltonian, for a few values of *N*, *N*′, see Eq. (18). (**d**) Dependence of *t*′, *v*′, and phase diagram of the winding number, *n*_*c*_, as a function of *N*, *N*′. In all panels *t* = 1.0, *v* = 1.5.

Each group of 2*N* sites is an effective ‘cell’ with two sub-lattices per cell (*i.e*. both DWs), just like in the standard bipartite lattice, but with just one single hopping *t*′. Therefore, the effective low-energy excitations are just like those in a monoatomic chain with period *N*, see Figs 1f and 3a. The basis functions, located at the DWs, at sites 2*mN*, (2*m* + 1)*N* are

11 $${\psi }_{a}^{\dagger }(m)=\alpha ^{\prime} \sum _{j=\mathrm{(2}m-\mathrm{1)}N}^{\mathrm{(2}m+\mathrm{1)}N-1}\,{(-\mathrm{1)}}^{j}{e}^{-\frac{\mathrm{|2}mN-j|}{\varepsilon }}{a}_{j}^{\dagger }$$

12 $${\psi }_{b}^{\dagger }(m)=\beta ^{\prime} \sum _{j\mathrm{=2}mN}^{\mathrm{(2}m+\mathrm{2)}N-1}\,{(-\mathrm{1)}}^{j}{e}^{-\frac{\mathrm{|(2}m+\mathrm{1)}N-\frac{1}{2}-j|}{\varepsilon }}{b}_{j}^{\dagger }.$$

the functions ${\psi }_{a}^{\dagger },{\psi }_{b}^{\dagger }$ are based in Eqs (5 and 6). While Eq. (5) is already symmetrically centered around 2*mN*, we need to multiply Eq. (6) by ${e}^{-\frac{1}{2}}$ to make it symmetrical around the DW at (2*m* + 1)*N*. The simple interpretation of this $\frac{1}{2}$ factor is that the center of symmetry is in the middle of two adjacent *b* sites, see the lower inset from Fig. 2.

The interaction between two localized states is, approximately:

13 $$t^{\prime} =\langle {\psi }_{a,k}|{H}_{pdw}|{\psi }_{b,k}\rangle $$

14 $$\approx \,\alpha ^{\prime} \beta ^{\prime} {e}^{-\frac{N}{\varepsilon }}[N(t{e}^{\frac{1}{2\varepsilon }}-v{e}^{-\frac{1}{2\varepsilon }})-(t{e}^{-\frac{1}{2\varepsilon }}-v{e}^{\frac{1}{2\varepsilon }})],$$

where the normalization constants *α*′, *β*′ are almost independent of *N*, $\alpha ^{\prime} \approx 0.87(1+\frac{1}{2}{e}^{\frac{-2N}{\varepsilon }})$. For large values of *N* the first term in the parenthesis dominates, but in some contexts -like in optics- the most common arrays consist of a limited number of waveguides.

The low-energy states given by Eqs (7) or (10), are no longer topologically protected, even though they are *locally* sub-lattice polarized (on a scale of *N* sites), but on a larger scale (*NM* sites) the sub-lattices are mixed. Also, their energy is genuinely finite, forming an s-band, Fig. 3a. This case was studied before in the continuum limit^26^ and the results agree with ours.

### New topological states and the bipartite lattice of DWs

In this section we introduce a bipartite lattice of domain walls, starting by its Hamiltonian, and derive its low-energy version, which is a new version of the SSH model, but with smaller hopping strenghts. As the SSH model, this new model has a phase with topologically protected edge states, see Fig. 3c. These new effective hoppings are strongly dependent on the distance between DWs, Fig. 3d. These predictions are confirmed by direct diagonalization of the full Hamiltonian, see Fig. 3b.

The distance between successive DWs can be dimerized, *i.e*. by setting the spacing from one DW to the next one as *N* cells to the right and *N*′ cells to the left. This changes slightly the Hamiltonian of a periodic lattice of DWs, from *H*_*PDW*_, Eq. (7) to the Hamiltonian of a bipartite lattice of DWs (BDW hereafter):

15 $${H}_{BDW}=\sum _{m=0}^{M-1}\,{h}_{N}(m)+{h^{\prime} }_{N^{\prime} }(m),$$

where the limits in the sums of *h*_*N*_′ ${h^{\prime} }_{N^{\prime} }$ must change accordingly. This produces two different hopping strengths between domain wall states:

16 $$t^{\prime} =\langle {\psi }_{a,k}|{H}_{BDW}|{\psi }_{b,k}\rangle \propto N{e}^{-\frac{N}{\varepsilon }}(t{e}^{\frac{1}{2\varepsilon }}-v{e}^{-\frac{1}{2\varepsilon }})$$

17 $$v^{\prime} =\langle {\psi }_{a,k}|{H}_{BDW}|{\psi }_{b,k+1}\rangle \propto N^{\prime} {e}^{-\frac{N^{\prime} }{\varepsilon }}(t{e}^{\frac{1}{2\varepsilon }}-v{e}^{-\frac{1}{2\varepsilon }}),$$

where, for simplicity, we dropped the last term of Eq. (14), this approximation is valid if $N,N^{\prime} \gg 1$ –see Fig. 3d.

The effective Hamiltonian for the BDW becomes a copy of the one for a bipartite lattice, but with a larger length scale and lower energies, see Fig. 3c,d:

18 $${H}_{BPD}^{eff}=\sum _{m=0}^{M-1}\,(t^{\prime} {\psi }_{a,m}^{\dagger }{\psi }_{b,m}+v^{\prime} {\psi }_{a,m+1}^{\dagger }{\psi }_{b,m})+H\mathrm{.}c\mathrm{.},$$

Figure 3b shows the energy spectrum of the full Hamiltonian *H*_*BDW*_. Inside the bulk gap there appears a band of the states at the DWs, and a new gap opens inside (reddish region), and in the middle of it two zero-energy states appear. These states are built from DW states, localized at the edges of the system and are fully sublattice polarized (see inset in Fig. 3b).

The analogy with the regular SSH model^24^ in the periodic case is almost complete, in the limit of an infinitely long chain, *M* → ∞. The Fourier transform of the effective Hamiltonian, Eq. (15), gives

19 $$h(k)=(\begin{array}{cc}0 & t^{\prime} +v^{\prime} {e}^{ik}\\ t^{\prime} +v^{\prime} {e}^{-ik} & 0\end{array}),$$

where *h*(*k*) is the kernel of the Hamiltonian. We can decompose *h*(*k*) as a linear combination of the Pauli matrices $\vec{\sigma }$ = (*σ*_*x*_, *σ*_*y*_, *σ*_*z*_),

20 $$h(k)=\overrightarrow{d}\cdot \overrightarrow{\sigma },$$

with $\vec{d}$ = (*t*′ + *v*′cos(*k*), *v*′sin(*k*), 0). Figure 3c shows the vector $\overrightarrow{d}$ for some values of *N*, *N*′, and keeping the original hopping strengths constant. The geometric place of this vector is a circle and we can define a topological invariant related to it, the winding number or Chern number:

21 $${n}_{c}=\frac{1}{2\pi }\oint dk\frac{{d}_{x}{d^{\prime} }_{y}-{d}_{y}{d^{\prime} }_{x}}{{d}_{x}^{2}+{d}_{y}^{2}},$$

the geometrical interpretation of *n*_*c*_ is very simple, it counts how many times the curve $\overrightarrow{d}$(*k*) encircle the origin. The two possible values of *n*_*c*_ = {0, 1} defines the phase diagram of the system^24^, see Fig. 3c,d. If *N* > *N*′ the system is in the ‘topological phase’, that is *n*_*c*_ = 1, conversely *N* < *N*′ is the trivial phase with *n*_*c*_ = 0. In the remaining case, *N* = *N*′, the curve $\overrightarrow{d}$ touches the origin and no topological index can be defined: the system became metallic. Only the case with *n*_*c*_ = 1 has topologically protected edge states.

While ${H}_{BDW}^{eff}$ is useful to visualize the connection of the BDW and SSH models, it doesn’t capture other interesting complex phenomena, such as disorder. In order to explore this, we will employ the full Hamiltonian, Eq. (15) in the next section.

## Disorder in the BDW Model

**Figure 4:**
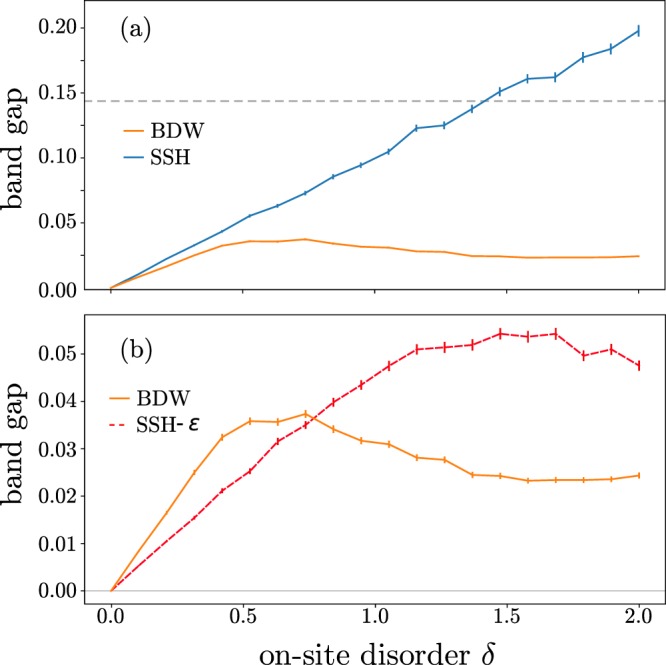
Average band gap between the edge states of the SSH and BDW models, as function of the width of the on-site disorder. The hoppings for the SSH and BDW curves are *t* = 1.0, *v* = 1.5 and *N* = 8, *N*′ = 14 for the BDW model (same total number of sites were used for the SSH model). The SSH-*ε* curve has *t* = 1, *v* = 1.1. The horizontal dashed line is the bandgap of the DW states (SSH bulk band gap is 1.0).

The resilience of topologically protected states in the SSH model to off-diagonal disorder is well-known, as well as its weakness against disorder on the on-site energies^27^. The band gap due to off-diagonal disorder is not shown since in the SSH model it is below our numerical precision. For the edges states of the BDW model, it lies below 10^−4^ for a disorder amplitude of *δ* = [0, 2*t*]. In smaller SSH chains the off-diagonal disorder is able to close the (residual) gap, but the chains considered in Fig. 4 are too large to show this effect.

The non-uniformity of on-site disorder directly breaks the chiral symmetry, destroying the topological protection. Figure 4a shows an almost linear band gap for the edge states of the SSH model.

The edge states of the BDW model are similar to their parent topological states, and they are –in principle– fragile to on-site disorder. However, the magnitude of the band gap due to on-site disorder is much smaller for the BDW edge states, see Fig. 4a. This can be partially explained as follows: While the SSH edge states are directly affected by the diagonal disorder, the BDW edge states are only affected by the averaged disorder over its characteristic length *ε*_*BDW*_ –which averages to zero for large *ε*_*BDW*_. In the Figure, the asymptotic-like band gap is well below the band gap of the bands formed by the DWs states, which in turn is much smaller than the bandgap from the SSH model. Given *t* = 1, *v* = 1.5, the characteristic length of the SSH model is ${\varepsilon }_{SSH} \sim 2.5$ cells, or about 5 sites. Instead –for the same hopping strengths *t*, *v*– the characteristic length of the SSH model, see Eq. (17), is ${\varepsilon }_{BDW} \sim 1.9$ supercells or 42 sites. To test the relationship between band gap and *ε*, one would naively compare a SSH and BDW chains with the same localization length of the edges states, by using different *t*, *v* in each chain. But, that comparison is unfair: while the SSH edge states are sub-lattice polarized, the BDW edge states also are sub-lattice polarized on the lattice of DWs. Therefore, one could expect a similar behavior of SSH and BDW chains when ${\varepsilon }_{BDW} \sim 2{\varepsilon }_{SSH}$, this is achieved when *t* = 1, *v* = 1.1 in the SSH and *t*′ = 1, *v*′ = 1.5 in the BDW model. Figure 4b shows similar band gaps for both models when the previous condition is satisfied.

**Figure 5:**
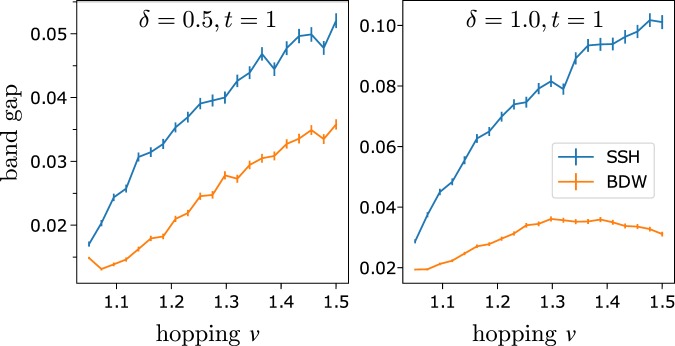
Average band gap between the edge states of the SSH and BDW models, as function of the hopping strength *v*. Different panels have a different on-site disorder amplitude *δ*. The hopping *t* = 1 for both panels, and *N* = 8, *N*′ = 14 for the BDW model (same total number of sites were used for the SSH model).

To understand the behavior of the BDW chain under on-site disorder, we show in Fig. 5 the averaged band gap when one of the hoppings, namely, *v* is varied, while keeping the other parameters fixed (*t*, *δ*). At moderate disorder (*i.e*. while the BDW increases linearly with disorder in Fig. 4a), *δ* = 0.5, both the SSH and the BDW models are similar: the band gap increases with *v*, which is to be expected since the localization lenght of edges states decreases with *v*. At each value of *v* the band gap of the BDW edge states is smaller than the gap from the SSH model: the BDW model has a band gap similar to the SSH with a smaller difference of the hoppings, *t* − *v*. Increasing the on-site disorder amplitude, *δ* = 1.0, Fig. 4b, the SSH bandgap is almost as twice as large for most values of *v*. But the BDW band gap doesn’t seem to increase appreciably: the resilience of BDW edge states to on-site disorder is a rather general feature and depends weakly of the original hopping amplitudes *t*, *v*. In contrast, the SSH model is fragile against disorder, especially when its hopping strengths are very different, that is, when *i.e. ε*_*SSH*_ comprises few sites.

**Figure 6:**
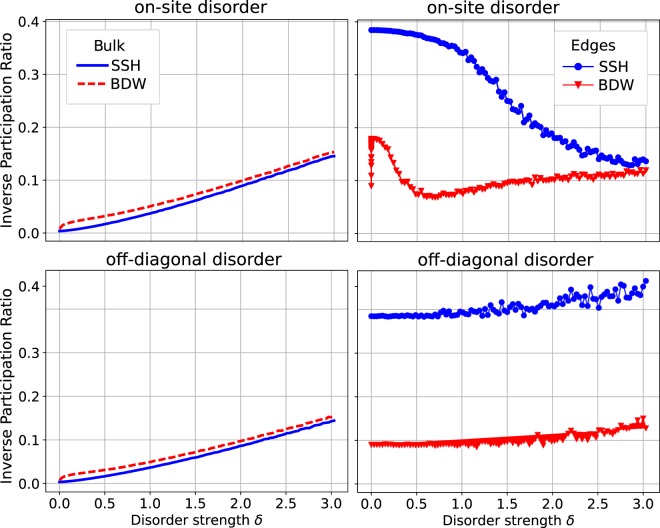
Averaged participation ratio as a function of the disorder strength, for on-site (upper panels) and off-diagonal disorder (lower panels). The bulk-like and edges states are in the left and right panels, respectively. The parameters used are the same as in Fig. 4.

To get a deeper insight on the effect of disorder on both, the SSH and the BDW models, we calculated the inverse participation ratio (IPR)^28^:

22 $$IPR=\frac{\sum _{n}|{c}_{n}{|}^{4}}{{(\sum _{n}|{c}_{n}{|}^{2})}^{2}},$$

where *c*_*n*_ is the wavefunction amplitude at site *n*. A completely localized state has *IPR* = 1, and a fully delocalized wave has *IPR* = 1/*N*, with *N* being the length of the chain. To introduce disorder into the SSH and BDW models, we add a random amount to the diagonal and/or the off-diagonal terms of the Hamiltonians. This random value is taken from a uniform random distribution of width *δ*. For diagonal disorder it is irrelevant whether the disorder averages to zero or not, since only differences in the on-site terms are meaningful. Figure 6 shows *IPR* for both, on-site and off-diagonal disorder (considering just nearest-neighbors). The Blöch states of the SSH lattice become localized with disorder, regardless of whether it is on-site or off-diagonal disorder. The regular Blöch states of the BDW have a very similar behavior -they are Blöch states too-, but they are slighty more localized for any finite value of *δ*. This is a consequence of the ‘fragmentation’ of the extended states (valence and conduction bands) into smaller groups with smaller bandgaps –see the green region in Fig. 3b. In the BDW model there is another type of Blöch states, these formed by the interaction between DW states, forming a wavepacket, see the green region of Fig. 3a,b. The *IPR* of these states is very similar to the regular Blöch states, and for clarity they are not included in Fig. 6.

The BDW’s edges states (right panels in Fig. 6) show a sudden jump of the *IPR* doubling its value for tiny amounts of on-site disorder. When *δ* is of the order of the interaction between the edges states, they no longer form an bonding anti-bonding pair, $\frac{1}{\sqrt{2}}({\psi }_{L}\pm {\psi }_{R})$, but instead they localize at the left or right edge. Due to the smallness of the energy involved in this process one can think of it as an artifact (*i.e*., the *IPR* should be ~0.18 for no disorder), but it shows that a very small on-site disorder can prevent the occurrence of charge fractionalization in the model^29^.

After reaching $\delta  \sim |t-v|$, the edge BDW states have a similar *IPR*, almost independent of the strenght of the on-site disorder *δ*: despite being localized by the chiral symmetry, the model allows a localization length of several sites, preventing a delocalization by on-site disorder. In contrast, the SSH edge states have an important delocalization due to the on-site disorder: the chiral symmetry already localized them to a few sites, and the breaking this symmetry overcomes the localization due to the disorder itself, the *IPR* decreases. This behavior, markedly different on both models, is consistent with the opening of a band gap by on-site disorder, Figs 4 and 5.

Finally, in regard to off-diagonal disorder, it increases the *IPR* of the bulk states of the SSH and BDW models. It also slightly increases the *IPR* of the edges states of both models. This is consistent with the absence of band gap due to off-diagonal disorder.

## Conclusions

We have examinated a generalization of the SSH model of polyacetylene to an array of domain walls focusing on the corresponding localized states. Under a mono-spaced lattice of domain walls an s-band appears in the middle of the bandgap, whose modes are formed by an extended superposition of the localized domain wall states.

If, instead, the domain walls form a bipartite lattice, a new bandgap appears in the middle of the extended domain wall states. This new configuration can host topologically-protected edge states, resembling the SSH model but on a much larger spatial scale and lower energies. A simple low-energy description was given, including the phase diagram of the system’s topological invariant.

The modes derived from domain walls states have interesting properties related to on-site and off-diagonal disorder. While in some aspects -such as the bandgap magnitude- they are more resilient to on-site disorder than SSH edge states, an almost negligible amount of disorder suffices to localize the wavefunction on a single edge -unlike the SSH edges states.

We believe that an understanding of the properties of low-dimensional systems in the presence of topological disorder (DWs) and local disorder (Anderson) is a necesary step towards the design of future robust low-loss devices. The effect of nonlinearity on the topological robustness of these systems is another subject of interest (under investigation) and will be reported elsewhere.
